# Back to Play Considerations in a Patient With Bell’s Palsy: A Case Report and Review

**DOI:** 10.7759/cureus.35739

**Published:** 2023-03-03

**Authors:** Bryce Grohol, Dillon Reno, Tanner J Jennings, Grayson T Fortin, Mark Rolfs

**Affiliations:** 1 Family - Sports Medicine, Liberty University College of Osteopathic Medicine, Lynchburg, USA

**Keywords:** psychological impacts, facial nerve paralysis, collegiate athlete, sports medicine, bell`s palsy

## Abstract

Bell’s palsy is an acute, ipsilateral facial paralysis secondary to inflammation of cranial nerve VII. This condition is classically caused by herpes simplex virus (HSV); however, many providers will make a diagnosis in the setting of other underlying conditions that are known to cause similar symptoms. The annual incidence of Bell’s palsy is 11.5-53.3 per 100,000 persons, with a small subset of individuals being contact sport athletes. A unique challenge to treating Bell’s palsy in collegiate athletes is finding a way for these players to return to their sport in a timely fashion, while also avoiding traumatic ocular injuries. Athletic goggles may provide a potential alternative option for athletes to return to the play of their respective sport prior to the physical symptoms subsiding. Due to the prolonged duration of most Bell's palsy symptoms, athletic goggles have the ability to save up to a full season of eligibility for a player. Aside from ocular injuries, a further challenge which encompasses all cases of Bell’s palsy is the negative psychosocial effects which accompany the physical symptoms of this condition. Both the patient's physical and psychosocial health considerations must be taken into consideration. In this case report, we review the utility of ocular protection in helping collegiate athletes with unilateral facial paralysis return to play prior to the resolution of symptoms.

## Introduction

Bell’s palsy, described in the 19th century by Sir Charles Bell, is associated with acute, ipsilateral facial paralysis. The diagnosis of Bell’s palsy is one of exclusion through a detailed patient history, physical examination, and laboratory or imaging studies. Although these symptoms are thought to commonly be due to an underlying herpes simplex virus (HSV) infection, facial paralysis has also been associated with neoplasms, trauma, infections, and iatrogenic causes [1]. Due to the vast spectrum of different etiologies, timely diagnosis and treatment are paramount for complete remission of symptoms.

The annual incidence of Bell’s palsy ranges from 11.5 to 53.3 per 100,000 persons across multiple populations [2]. Symptoms can range from mild to severe facial paralysis and can manifest as impaired blinking, decreased protection of corneas, impeded speech, and hindered smiling [1]. Patients may also experience ear pain, change in taste, dry eyes, epiphora, and hearing disturbance. Most Bell’s palsy symptoms peak within 72 h [1-3].

A specific subset of individuals who experience Bell’s palsy are contact sport athletes. In this patient population, Bell’s palsy presents a unique hurdle for sports medicine physicians due to the difficulty associated with safely returning the athlete to their respective sport. The National Collegiate Athletic Association (NCAA) allows only four seasons of eligibility for student athletes who do not complete a red shirt year, meaning that the four-to-six week average duration of symptoms is enough to sideline a player for a significant portion of their athletic career [4]. Currently, there is no well accepted or effective option to help these athletes return to play within a reasonable time frame. In this report, we detail an uncommon scenario and novel treatment option of a young college athlete who presents with acute onset unilateral facial paralysis. This patient must navigate treatment options which allow him to return to play without sacrificing mental health.

## Case presentation

An 18-year-old collegiate men’s soccer player presented to the student health clinic with acute onset left-sided facial paralysis, which began upon awakening that morning. He denied any other neurological symptoms and had no pertinent past medical history.

His vital signs were in normal range (blood pressure: 122/88; pulse: 49; respiratory rate: 16; SpO2: 97%; temperature: 99 degrees Fahrenheit), and he appeared in no acute distress. His gait and station were normal, however, cranial nerve testing revealed facial droop and an inability to smile or raise his forehead on the left side of his face. Upper and lower facial paralysis on the left side was evident. There was no excessive lacrimation or drooling, and there was no change in hearing or taste. Gross sensation and monofilament test were intact. Deep tendon reflexes were 2+ bilaterally throughout and the finger-to-nose test was intact. Romberg’s sign was negative, and he showed no tremors. Muscle tone and strength were both 5/5 bilaterally.

The patient was diagnosed with Bell’s palsy and started on valacyclovir 1 g by mouth once a day for seven days, along with a prednisone taper for management of symptoms. The patient was instructed to return for care if his condition worsened.

The following day, the patient was evaluated at the university athletic training facility by an athletic trainer and the team physician. The examination by the team physician found that the patient was able to close his left eye, but only with significant effort. This along with the other facial symptoms supported the diagnosis of Bell’s palsy. 

Due to the risk of eye injury, the team physician deemed it unsafe for the patient to participate in men’s soccer until he regained the ability to completely close his left eye with ease. Alternatively, however, while the athlete waited for his facial paralysis to resolve, it was suggested that wearing protective eyewear may allow him to return to play without missing time. An eye patch was discussed as a possible solution; however, due to its negative impact on depth perception, a pair of goggles was ultimately decided upon and ordered for the patient. The goggles came with an adjustable elastic strap as well as scratch resistant lenses. While this specific model was not air-tight, it did include foam lining around the circumference of the plastic frame to maximize comfort for the wearer. When the patient tried on the goggles, he felt self-conscious with his appearance and made the decision not to wear them. Consequently, the patient was held from men’s soccer while his symptoms subsided, rather than returning to play immediately while wearing the goggles.

## Discussion

Bell’s palsy is described as an acute unilateral paralysis of the face, with 70% of patients experiencing complete paralysis [2-3]. The etiology of Bell’s palsy commonly involves inflammation and ischemia of cranial nerve VII, otherwise known as the facial nerve, due to compression of the nerve through the geniculate ganglion inside the facial canal [5]. Although it usually resolves in weeks to months, Bell’s palsy can potentially lead to long term issues with eyelid closure which increases the risk of permanent eye damage [6]. For athletes with university scholarships, being held from play while it resolves may not be a viable option.

With a lifetime risk of 1 in 60, the median onset of Bell’s palsy is roughly 40 years of age, however, it can be seen in all age groups. Risk factors for Bell’s palsy include pregnancy, preeclampsia, obesity, and hypertension [5]. Bell’s palsy is classically described as being idiopathic, however, many healthcare providers will diagnose Bell’s palsy in the setting of other underlying conditions which are known to cause unilateral facial nerve paralysis. Table 1 shows several of the most common underlying causes of Bell's palsy [1, 5, 7].

**Table 1:** Known causes of facial paralysis. [1, 5, 7]

	Causes of facial paralysis
Infectious	Herpes simplex virus, Lyme’s disease, otitis media, influenza
Malignancy	Facial neuroma, acoustic neuroma, geniculate hemangioma, parotid neoplasm
Traumatic	Forceps delivery, bone fracture, penetrating injuries
Iatrogenic	Post-surgical
Idiopathic	Sarcoidosis, Guillain-Barre

The diagnosis of Bell’s palsy is one of exclusion and although there is no specific time requirement for onset, symptoms begin quickly and typically peak within 72 h [8]. Nasolabial folds may disappear, the forehead can unfurrow, and the corners of the mouth often droop in affected patients. Eye irritation commonly develops due to the inability to completely close the affected eye [8]. The patient described in this case report manifested these symptoms rapidly. The House Brackmann Scale, while not utilized by the provider in this instance, is a commonly used grading system which objectively analyzes the degree of facial nerve dysfunction in affected individuals. The scale ranges from I (normal) to VI (total paralysis) in patients suffering from Bell’s palsy [9]. This scale can be used to communicate the severity of paralysis regardless of the underlying cause of the condition.

The American Academy of Neurology (AAN) recommends treating all Bell’s palsy patients with oral corticosteroids to maximize the probability of complete recovery of the facial nerve. Oral antiviral therapy, although not proven to be beneficial, is also commonly prescribed in combination with corticosteroids due to the high suspicion of HSV infection as an etiology [10]. This patient was given a prednisone taper within 72 h of symptom onset and started on a course of oral valacyclovir. Although the standard treatment of Bell’s palsy includes both of these agents, newer data have shown no significant difference in recovery with the addition of antivirals to the steroid regimen [10-11]. Table 2 highlights the details of pharmacologic therapy in the treatment of Bell's palsy [7, 12]. Alongside pharmacologic treatment, physical therapy has been shown to provide some benefit in patients with Bell's palsy. One review reported that patients with facial paralysis who underwent physical therapy in addition to standard drug therapy had both an increase in functional recovery as well as a decreased time to complete recovery compared to standard drug therapy alone [13]. Physical therapy could potentially be used in athletes alongside the proposed treatment strategy in this article. 

**Table 2:** Pharmacologic interventions for the treatment of Bell's palsy. [7, 12]

Treatment	Dosing regimen	Adverse reaction
Acyclovir	Adults: 400 mg five times daily for seven days; Children over two years: 80 mg per kg daily divided every six hour for five days (maximum daily dose 3200 mg)	Gastrointestinal upset, headache dizziness, elevated liver enzymes
Valacyclovir	Adults and children over 12 years: 1 g three times daily for seven days	Gastrointestinal upset, headache dizziness, elevated liver enzymes
Prednisone	Adults: 60 mg daily for five days, then 40 mg daily for five days	Headaches, insomnia, weight gain, hyperglycemia
Artificial tears	Adults and children: place drops four to six times daily in affected eye for corneal protection	Blurry vision, eye irritation

While not utilized in this case, an osteopathic manipulative treatment (OMT) is another modality that can be considered in the management of athletes with Bell’s palsy. Considerations to cranial, lymphatic, cervical, and sacral somatic dysfunctions with subsequent osteopathic manipulative corrections have been the focus of several case reports [14-15]. Specifically, cranial sacral OMT has been postulated to relieve pressure on cranial nerve VII, thus reducing the severity and duration of Bell's palsy symptoms arising from the facial nerve [14]. Furthermore, enhancing lymphatic flow can also be used to expedite the recovery of Bell’s palsy, particularly by focusing OMT on restrictions found in the respiratory diaphragm, thoracic outlet, sub-occipital diaphragm, and cerebellum tentorium [15]. 

Bell’s palsy symptoms typically stabilize after approximately three weeks then gradually improve by two to three months. Although many patients experience a full recovery, some patients see lasting adverse effects. Roughly 30% of those diagnosed are left with some degree of permanent facial asymmetry [8]. This long-term disfigurement, due to inadequate recovery of the facial nerve, has been shown to disrupt quality of life by impairing interpersonal relationships and leading to social distress, depression, and isolation [16].

An essential precaution in patients with Bell’s palsy, especially athletes, is ocular protection. Due to Bell’s palsy causing an inability to close the affected eye, the risk of traumatic eye injury increases substantially [6]. With nearly a third of all eye injuries in the United States being related to sports, athletes are already at an increased risk [17]. Prevention is the most important aspect of treating orbital injuries, and protective eyewear has been shown to reduce the incidence of serious injuries in athletes [18-19]. Contact sport athletes are typically held out of participation of sports in instances such as this to completely eliminate the risk of trauma to the eye. Although Bell's palsy is considered a transient condition, the duration of symptoms is unpredictable and can last for extended periods of time. This can result in loss of multiple games if not entire seasons of eligibility for the affected athlete. While this method is effective in reducing the risk of harm, it is not acceptable to many players with limited time left in their athletic careers.

Studies have shown that up to 90% of sports-related ocular injuries could have been prevented with appropriate eye wear, including sport goggles [17]. The NCAA has very few recommendations regarding eye protection for collegiate athletes. One organization which the NCAA has chosen to endorse, the American Society for Testing and Materials (ASTM), has set standards for ocular protection in different sports [17]. The guidelines describe the requirements for specific goggles and spectacles which they consider adequate for protecting against ocular injury in athletes. These approved devices can withstand balls or other similar projectiles fired at a rate of 90 miles per hour and commonly have polycarbonate frames. The ASTM has specific protocols which they have modified to apply to many popular sports [17, 20]. While the benefits of sports goggles are well documented in protection against injury, it has been shown that only 15% of youth athletes who participate in organized sports utilize them [20]. In the case described, an eyepatch was originally suggested; however, due to the limitations on depth perception, a pair of goggles was found to be a more suitable alternative. Due to the negative self-perception of Bell’s palsy as well as the inconvenience of the goggles, the patient elected to refrain from athletics for the entire duration of his symptoms rather than be subjected to the emotional strain that came with his condition and the proposed solution. Physical protection against trauma, such as athletic goggles, has the ability to save an athlete from extended periods of missed time and loss of eligibility. Ocular protective measures, such as ASTM approved eyewear, should be considered for contact sport athletes with facial paralysis.

## Conclusions

Bell’s palsy is a unique condition that often resolves spontaneously over weeks to months. Oral corticosteroids are well accepted as treatment to decrease the duration of Bell’s palsy symptoms. Antiviral agents, while commonly used, have inconsistent results with large variations in reducing overall recovery time between individual patients. For athletes, specifically at the collegiate level, additional treatment may come with use of athletic goggles for ocular protection. The prolonged recovery period of Bell’s palsy is less than desirable due to sports eligibility being limited to only four years. To decrease the risk of ocular injuries in these athletes while also allowing them to return to their respective sports in a timely fashion, adequate eye protection can be considered. This added protection, although potentially effective, can further contribute to the already negative effects Bell’s palsy has on mental health and quality of life. Our objective with this case report is to bring further attention to the management of Bell’s palsy with respect to ocular injury risk and mental health. We believe that athletic goggles, although somewhat stigmatized by the athletes themselves, provide an alternative option to expedite the athlete's return to play. It is our opinion that protective eyewear should be considered in all cases of Bell's palsy which occur in contact sport athletes. Further studies should be considered to investigate alternative solutions which not only offer adequate eye protection for athletes, but also limits the negative social effects already present with facial asymmetry.
